# Research on Expressive Writing in Psychology: A Forty-year Bibliometric Analysis and Visualization of Current Status and Research Trends

**DOI:** 10.3389/fpsyg.2022.825626

**Published:** 2022-10-13

**Authors:** Xiaojuan Gao

**Affiliations:** Foreign Language Department, School of Law & Humanities, China University of Mining and Technology-Beijing, Beijing, China

**Keywords:** expressive writing, research trends, positive psychology, psychology, bibliometric analysis, visualization

## Abstract

This study offers a systematic review of global academic publications of studies on expressive writing in psychology to date. By using two visualization tools VOSviewer and CiteSpace, we analyzed 1,429 articles on expressive writing from the Web of Science (WoS) Core Collection database. This study might be the first attempt at providing a visualized analysis of the existing expressive writing research. It discusses the results from the following three aspects: (1) the descriptive analysis of general results based on publications, (2) the content analysis based on highly cited articles and keyword analysis, (3) the thematic evolution based on co-word analysis and bursts detection. It is found that the application of expressive writing to minority ethnic groups might be one of the future research interests. The study proposes the necessity of conducting research in the context of positive psychology, argues for a combined use of creative and expressive writing in future studies, and suggests the potential of second/foreign language expressive writing research. The study can be used to enhance researchers’ understanding of expressive writing research and provide insights into future research opportunities in this area.

## Introduction

Expressive writing as a way of written emotional disclosure can enhance people’s psychological and physical health outcomes (Pennebaker and Beall, 1986; Liebman, 1992; Smyth, 1998; Lepore et al., 2002). Pennebaker and Beall (1986) pioneered the research area by investigating the relationship between expressive writing and health reports in “Confronting a Traumatic Event. Toward an Understanding of Inhibition and Disease” published in *Journal of Abnormal Psychology*. In the study, 46 healthy undergraduate student participants were randomly assigned to write one of the four topics for 15 min each day, on four consecutive days. The control group wrote on trivia topics; the trauma-emotion group wrote about feelings related to traumatic events; the trauma-fact group wrote only facts about the events and the trauma-emotion-fact group wrote about both feelings and facts. The results show that the trauma-emotion-fact group reported better immune system, improved mood, and had fewer health center visits in the following 6 months after the experiment. The contribution of this study is that it initiated an expressive writing paradigm for numerous subsequent studies by other scholars.

Since 1980s, a plethora of studies have shown that expressive writing is a beneficial method to address traumas and emotional upheavals in a healing way (Pennebaker and Evans, 2014). This method has shown positive results on people’s health and psychology on multiple levels, such as in cognitive, emotional, social, and biological aspects (Pennebaker, 2004). For example, expressive writing is efficacious for people’s health, and they have fewer visits to health centers in the subsequent months after the writing intervention (Pennebaker and Beall, 1986; Burton and King, 2004). Concerning psychological outcomes, expressive writing is beneficial for reducing anxiety (Sexton et al., 2009; Pennebaker and Chung, 2014), mediating symptoms of depression (Gortner et al., 2006), relieving post-traumatic stress (Pavlacic et al., 2019), among other benefits.

Ever since the first paper on expressive writing was published in 1980s, thousands of subsequent studies have explored effects of expressive writing on human beings in terms of physical or psychological outcomes. The past three decades, especially after the year 2000, have witnessed the rapid development in the use of expressive writing in different fields for theoretical explorations and empirical research. With the rapid increase of the publications of the scientific literature, keeping abreast of large volumes of academic literature data of such divergent research areas and themes where expressive writing is used would pose significant challenges even to diligent and active researchers.

The most widely used method to review the written literature in this field is meta-analysis. However, to the best of our knowledge, ever since the wide use of data mining, no research has globally investigated the academic publications on expressive writing in psychology using visualized bibliometric analysis. Hence, the evolution of the area, the thematic focus, the research hot spots, and the trends in this area remained unclear.

Bibliometrics is a quantitative analysis method of written literature (Ellegaard and Wallin, 2015; Ninkov et al., 2021). It is to analyze bibliographic features of the publications (e.g., abstracts, keywords and citations) using multiple mathematical and statistical techniques in a given research field and effectively summarize the characteristics of publications and show relationships between the published works (Broadus, 1987). Besides, bibliometric method can describe developmental trend of a certain field and clarify the characteristics of the current literature. This method has been used extensively in academic research in various fields, such as in environmental studies (Zhang et al., 2015; Lu et al., 2021), management (Ali et al., 2019), education (Diem and Wolter, 2013), linguistics (Meara, 2012; Wang et al., 2019), and culture studies (Peng et al., 2021). In psychology, only a few researchers have conducted research using this method, and mostly focusing on publication or research trends of certain journals (Piocuda et al., 2015), very rare cases focusing on certain research topics, only one found in the discussion of postpartum depression (Bai et al., 2021). Therefore, it seems essential to introduce this method to explore research topics in psychology so that on the one hand emergent researchers could efficiently grasp the overall landscape of a given field, and on the other hand, mature researchers might gain insights inspirations from a wholistic perspective.

Since bibliometric methods can effectively synthesize and analyze a large number the existing literature, and this study is likely to be the first attempt at providing a quantitative analysis of the thousands of publications on expressive writing in psychology. It is essential to summarize the studies available and visualize the knowledge domain, in the hopes of drawing a clear bibliographic landscape of the related studies. Conventional literature reviews may require a scholar to read every article in the field and make subjective and personal judgments after having finished reading a large volume of literature, while bibliometrics can adopt big data as a method to provide a comprehensive and objective results of the existing literature.

In bibliometrics, visualization is a common practice using scientific knowledge mapping tools, such as CiteSpace, VOSviewer, Gephi, and BibExcel. Among them, CiteSpace and VOSviewer are the most widely used because of their multiple functions, simple operation, and eye-appealing mapping output.

The aims of the study were to (1) quantitatively and qualitatively demonstrate the current research status regarding expressive writing, including the overall publication trend, national/regional distributions, influential authors, journals and institutions, most cited literature, (2) identify major research topics and research hotspots, (3) show development trends using topic modeling and discuss future directions of the field.

The study has three contributions. Firstly, it is the first visualized analysis of expressive writing research, which makes it accessible to researchers outside the field to know the current status of the existing scholarship. Secondly, it shows the major fields in which expressive writing has been applied, offering potential academic guidance for emergent scholars. Thirdly, it lists the most influential journals, institutions, and references in the field and discusses ongoing hot topics, which will assist future interested scholars in continuing the discussion. Besides, it posits more possibilities of using expressive writing among bilingual or multilingual speakers and the value of situating related studies in the context of positive psychology.

## Methodology

### Data Collection

#### Data Source and Search Strategy

In order to ensure the scientific integrity of the data source and reliability of the research conclusions, bibliographic records of the published literature were collected from the core collection of the Web of Science (WoS) database of Thomson Reuters Scientific. WoS is an inclusive database that covers virtually all the important databases in arts and science and includes almost all the bibliographic information of the high-quality research papers. Besides, Web of Science database is a preferred source of data for bibliometric analysis because of its multi-disciplinary data, wide range of literature, and completeness of the bibliographic information (Falagas et al., 2008; Archambault et al., 2009). WoS Core Collection used in this study includes 10 databases: Science Citation Index Expanded (SCI-EXPANDED), Social Science Citation Index (SSCI), Arts and Humanities Citation Index (A&HCI), Conference Proceedings Citation Index-Science (CPCI-S), Conference Proceedings Citation Index-Social Science and Humanities (CPCI-SSH), Book Citation Index-Science (BKCI-S), Book Citation Index-Social Science and Humanities (BKCI-SSH), Emerging Sources Citation Index (ESCI), Current Chemical Reactions Expanded (CCR-EXPANDED), and IC (Index Chemicus).

In order to retrieve literature comprehensively and accurately on expressive writing, a combination of retrieval strategies was used in data collection. For this study, the search strategy was set as follows:

Topic = (“expressive writing” OR “written disclosure” OR “expressive disclosure” OR “written emotional expression” OR “written exposure therapy” OR “written expressive disclosure” OR “writing intervention” OR “writing therapy” OR “disclosure of trauma*” OR “writing about traumatic event*” OR “emotional disclosure” AND “writ*” OR “disclosure” AND “writing”) which means that literature with these words or phrases in title, or abstract or keywords will be retrieved.

A total of 1,809 articles were retrieved, with the time span set from 1981-01-01 to 2021-12-31.

#### Data Selection and Exclusion Criteria

With 1,809 results shown, the data was filtered by ‘Document Types = ("Articles" OR "Review Articles"),’ obtaining 1,535 documents left. In this study, only research articles written in English were included to analyze, and we gained 1,464 bibliographic records. Then, we went through every acquired article to check the relevance of the results and did manual exclusion of 35 results because the topic and content were not in line with the purpose of the paper. For instance, some studies were the public reports of the cardiology foundation and their practice guidelines and therefore were deleted. Duplications and retreats of publications were also removed. Eventually, a total sample of 1,429 publications that met all the criteria was independently exported from WoSCC to plain text file containing “Full Record and Cited References”. That is to say that the bibliographic database itself is a text file that includes various variables, such as title, author, abstract, keywords, references, and other related information. The investigated sample includes 1,333 articles and 96 review articles, covering a wide range of WoS categories, such as “psychology clinical,” “education,” “linguistics,” “psychiatry,” “rehabilitation,” “Neurosciences,” “nursing,” “literature,” and “communication.” These articles represented almost all of the high-quality studies on the evolved topic “expressive writing” around the world from 1980s till 2021.

**Figure 1:**
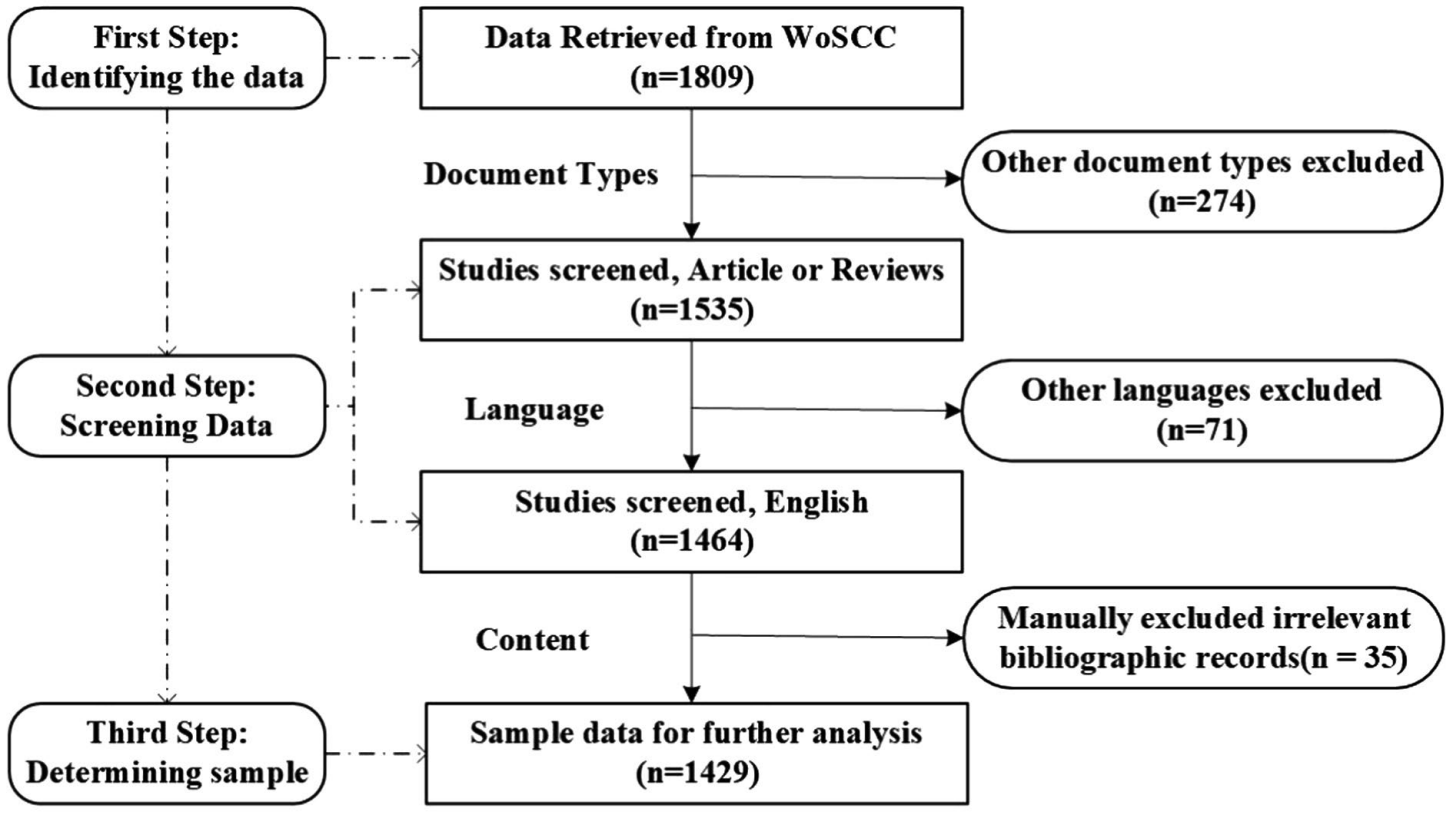
The flowchart of retrieving expressive writing publication bibliographies. WoSCC, Web of Science Core Collection.

Table 1 displayed the summary of the data collection details and retrieval strategy except keyword searching strategy. Figure 1 shows the flowchart of the retrieval process of the published literature in this study.

**Table 1:** Summary of data collection and search strategy.

Data source	Web of science core collection
Database	SCI-EXPANDED, SSCI, A&HCI, CPCI-S, CPCI-SSH, BKCI-S, BKCI-SSH, ESCI, CCR-EXPANDED, IC
Searching period	From 1981-01-01 to 2021-12-31
Document types	“Articles” or “Review Articles”
Language	“English”
Sample size	1,429
Date of retrieval	7 February 2022

### Analysis Method and Tools

#### Bibliometric Analysis

In the present study, the bibliometric analysis function of Web of Science and Microsoft Excel were both adopted to display the basic statistical indicators of the publications in terms of annual publications, country/region distributions, influential authors, major institutions and their cooperation. Their cooperation between these indicators was also analyzed to reveal the overall landscape of the retrieved studies. Co-cited authors and co-cited articles were also obtained to exhibit the intellectual base and strength of the field.

#### Content Analysis

Content analysis is inferred from the keywords and abstract of the literature based on quantitative statistical analysis. To be specific, keyword analysis is extracted from the author keywords, keyword plus offered by WoS, and keywords extracted from the abstracts based on equation results of bibliometric analysis. Thus, keyword analysis, like topic modeling, is a tool in bibliometrics to do content analysis to reveal characteristics, research hotspots, changes in research directions, and future research trends in a given field. In addition, co-word analysis technique, one of the most important and widely used tool for content analysis, is to extract topic-related terms from titles, keywords, and abstracts of the publications to identify research focus and track research trends from different perspectives (van Eck and Waltman, 2009). In the present study, most cited references or highly cited article analysis, keyword analysis, and co-word analysis were all employed as the primary methods for content analysis to discover and identify the current research status and predict future tendencies of expressive writing studies.

#### Data Visualization

This study uses two java-based visualized analysis tools, CiteSpace and VOSviewer, to perform the visualization results of the relevant bibliographic records to form corresponding knowledge mapping. CiteSpace is a software designed to detect and visualize “emerging trends and transient patterns in scientific literature” (Chen, 2006). It is able to track the dynamic progress of a scientific field based on data mining (Chen, 2006; Li and Chen, 2016). The software utilizes embedded mathematical equations to calculate quantitative results of a large number of bibliographical records, visualize multiple co-citation and co-occurrence networks regarding major authors, institutions, countries/regions, and references to reveal the underlying connections between them (Chen, 2006). CiteSpace can also slice the given period of publications to generate a knowledge map of the timeline view or time zone view of the research focus and thematic progression. In this study, CiteSpace (version 5.8.R3) was used for collaboration network analysis, co-citation analysis, and the calculation of high-frequency keywords. VOSviewer is another publicly available knowledge mapping software developed by Nees Jan van Eck and Ludo Waltman from Leiden University, in the Netherlands (Waltman et al., 2010). The software can produce bibliometric maps freely in an easy-to-interpret manner based on the data of co-authorship, co-occurrence, co-citation, and bibliographic coupling, among others. In this study, VOSviewer (version 1.6.18, Leiden University, Netherlands) was used to process co-occurrence analysis and generate topical keywords clustering map to uncover hot research topics and areas using its clustering techniques and data mining function. In both bibliometric tools, the size of nodes and density of the clustering represent the significance and level of impact of the relevant literature. The width of lines and distance between nodes symbolize the strength of connections.

## Results

### Trend of Annual Number of Publications

**Figure 2:**
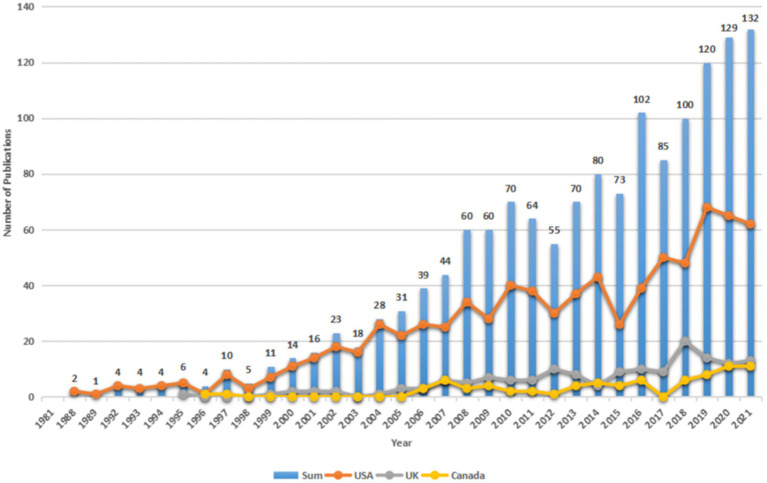
Number of yearly publications, and growth curves in the top 3 countries in the studies of expressive writing during 1981–2021. The bar represents the annual number of total publications worldwide. The legends of the United States, the United Kingdom, and Canada represent that they are the top 3 most productive countries and their performance was illustrated by the three curved lines overlayed on the charts.

The annual publication distribution for expressive writing in psychology is demonstrated in Figure 2. It is observed that a majority of publications were produced in the past decade and that the field is now witnessing blossoming in research output. The result suggests that an increasing number of researchers have started to direct their attention to this field over the last decade.

The growing pattern and its corresponding chronological distribution show that there were two major stages in the publication trend: before the year 2000 and after 2000, which can be regarded as the preliminary stage and the growth stage. Before 2000, in the 1980s, although we set the starting year in 1981, the first research output under the search strategy appeared in 1988, and in 1990s, only a small quantity of articles were published and the number increased slightly with fluctuations. From 2000 onward, the annual number of publications has been on a steady rise till 2010. Between 2010 and 2021, there were fluctuations, but since 2018, the number of articles stood over 100 and rose steadily to 132 in 2021. After the sharp rise to 102 articles in 2016, the number of publications in the past 6 years accounted for almost half of the total publications (46.7%). It can be observed that the research area has been drawing tremendous attention in the past two decades and the expressive writing publications are expected to continue to be on the rise in psychology and other disciplines in the coming years (Figure 2).

### Cooperation Network Analysis

#### Highly Contributive Countries/Regions, Institutions, and Their Cooperation

The number of publications by each country and their citations suggest the impact of the most productive countries in the focal field of expressive writing in psychology. The 1,429 published articles on expressive writing were produced by 69 countries/regions apart from 11 records (0.75%) that do not contain related data. Out of a total of 69 countries involved, 11 countries (15.9%) produced more than 20 papers, and 14 countries (20.3%) have more than 200 citations. Thus, the analysis here includes countries with at least 20 papers and 200 citations. The result of this analysis shows that the 10 most productive countries/regions include the top 1 highly contributive country United States, followed by the United Kingdom, Canada, Australia, Netherlands, Germany, Italy, China, Switzerland, and Israel (see Table 2). The top 10 countries contributed to 95.17% (1,360 articles) of the total publications. It is observed that most countries in the top 10 list are from North America and Europe and that the only country in the Asia–Pacific region is China. The number of publications from the United States (799 publications) has surpassed the sum of the rest of the nine other most productive countries (561 publications), which shows that the United States has taken a leading position in conducting research on expressive writing. The highest number of citations were also obtained by the United States, followed by the United Kingdom. In terms of average citations per article, the rate was distributed different than the other dimensions. For instance, United States, Netherlands, and Australia occupied the top 3 positions.

**Table 2:** The top 10 contributive countries/regions.

Rank	Country/region	TP	PTP (%)	TC	AC	LS
1	United States	799	55.91	26,858	33.61	117
2	United Kingdom	180	12.60	2,492	13.84	81
3	Canada	78	5.46	1,279	16.40	36
4	Australia	59	4.13	1,104	18.71	21
5	Netherlands	55	3.85	1,526	27.75	29
6	Germany	49	3.43	645	13.16	23
7	Italy	49	3.43	802	16.37	13
8	P. R. China	36	2.52	327	9.08	23
9	Switzerland	28	1.96	385	13.75	23
10	Israel	27	1.89	375	13.89	9

TP, Total publications; PTP, Percentage of the total publications; TC, total citations; AC, Average number of citations per publication; LS, Link Strength in co-authorship. The data of United Kingdom includes merged data of England (157 articles), Scotland (12 articles), Wales (10 articles), and Northern Ireland (four articles), while the data of the respective regions remained unchanged in the mapping.

**Figure 3:**
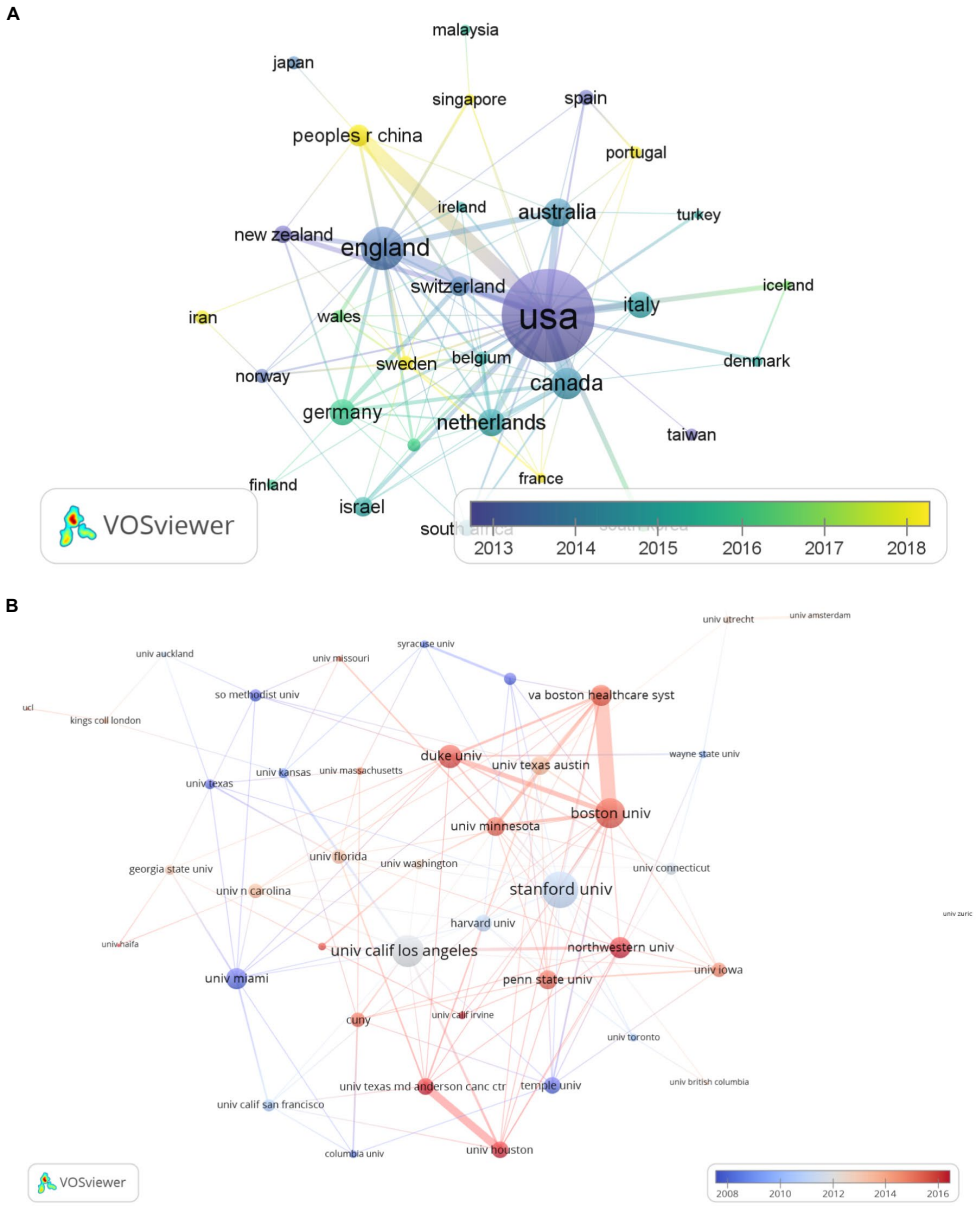
**(A)** The cooperation network of the major countries/regions engaged in expressive writing studies. **(B)** The cooperation network between institutions (node label threshold ≥10). The size of nodes (i.e., institutions) denoted the published documents number, and the warm color suggested that the cooperation years were more recent than the ones illustrated by the cold colors. The width of the connecting lines represented the strength and frequency of the collaboration between two institutions.

According to Figure 3A, the collaborations in the expressive writing studies between different countries/regions were very frequent. The degree of the cross-national cooperation is evaluated by the number of jointly published articles by two countries/regions. In VOSviewer, the visualization was realized by the co-authorship function together with parameters in the scale and layout function in the visualization. The size of the nodes represents the average number of published articles per year, with a larger node representing countries/regions with more internationally co-authored articles. The strength of the connecting lines between two countries/regions indicates the frequency of the collaboration between them. The United States, the United Kingdom, and Canada ranked the top three in their academic cooperation with other countries/regions. The United States has the largest node and its connections with China, England, Australia and Switzerland, New Zealand, and Netherlands are frequent and strong. Figure 3A reveals that the geographical location does not seem to affect researchers when they seek international cooperation in conducting studies in the field.

There were 1,287 institutions contributing to the studies in expressive writing. University of Texas at Austin has the largest number of publications (35 articles), followed by University of California, Los Angeles (34 articles), Boston University (34 articles), and University of Houston (25 articles). The University of Texas at Austin had the highest number of citations (2,834 times), followed by University of Texas (2,669 times; Table 3). Other universities that have total citations of over 1,000 times are SUNY Stony Brook University (1,798 times), South Methodist University (1,629 times), University of California, Los Angeles (1,246 times), Columbia University (1,203 times), University of California, Riverside (1,015 times) although a few of these universities have less publications. It can be observed in Table 3 that all the highly contributive institutions are located in the United States. The imbalance in geographical locations reflects the leading role of American institutions in publishing research on expressive writing, and shows the future potential of using expressive writing in different cultural backgrounds.

**Table 3:** The top 11 most contributive institutions.

Rank	Institution	TP	PTP (%)	TC	AC	LS
1	University of Texas at Austin	35	2.45	2,834	80.97	38
2	University of California, Los Angeles	34	2.38	1,246	36.65	65
3	Boston University	34	2.38	646	19.00	118
4	University of Houston	25	1.75	344	13.76	50
5	University of Texas	23	1.61	2,669	116.04	21
6	University of Texas MD Anderson Cancer Center	23	1.61	161	7.00	44
7	Wayne State University	22	1.54	736	33.45	24
8	University of Minnesota	21	1.47	306	14.57	57
9	VA Boston Healthcare System	20	1.40	350	17.50	78
10	Pennsylvania State University	19	1.33	468	24.63	33
11	Duke University	19	1.33	403	21.21	49

TP, Total publications; PTP, Percentage of the total publications; TC, total citations; AC, Average number of citations per publication; LS, Link Strength in co-authorship.

Figure 3B displays the academic cooperation between institutions. The threshold was set 10 for the minimum number of publications of each institution, and a total of 43 institutions met the thresholds in visualization.

The academic cooperation between the institutions mainly took place between two groups, one being Boston University, Duke University, University of Minnesota and VA Boston Healthcare System, and the other University of Houston and University of Texas MD Anderson Cancer Center. The warm-color strong connecting lines between them also show that their cooperation started in the past few years and their cooperation would be very likely to continue in the future. It can also be observed that University of Texas, University of Texas at Austin, and University of California Los Angeles was the main center of collaborations in the past but not in recent years. Besides, the cooperation network between institutions suggests that geographical location might be a consideration in conducting collaborative research, and the strong cooperation between university and research centers can be seen as a common pattern in doing research related to expressive writing.

Compared with cooperation between countries, the institution cooperation network was surprisingly different since the exchange between cross-border institutions seems deficient and the collaboration mainly takes place inside the United States. Future international collaborations among institutions across countries could be strengthened. This result could be useful for international researchers to find influential research institutes and potential research teams to work with. So that a more diversified global expressive writing community can be established.

To conclude this part, the United States has published the most articles and has the highest number of citations, showing that it has a significant and leading role in the field. The United States was also occupying the main position in international collaboration network in terms of country cooperation. However, the American academic institutions still need to promote its academic communication and cooperation with their counterparts in other parts of the world. Meanwhile, Asian countries and institutions were underrepresented in this field and more research outcomes in expressive writing are expected from this region.

#### Highly Contributive Authors and Their Cooperation

Scholars with highly cited publications often lead the research trends of an area, and identifying the most influential scholars could show the readers who have led the research trends in the field. Besides, tracking the publications of these scholars could help the emergent scholars save time to understand the research focus and future directions. The result of this study shows that 4,084 authors contributed to the 1,429 publications. With a combined use of the bibliometric statistical results provided by WoS, together with VOSviewer author citation statistics, the highly contributive authors in the research of expressive writing are exhibited in Table 4.

**Table 4:** The most influential authors in expressive writing studies.

Rank	Author	Current affiliation	TP	PTP (%)	TC	AC
1	Pennebaker, James W.	University of Texas at Austin	40	2.80	9,081	227.03
2	Sloan, Denis M.	VA Boston Healthcare System	26	1.82	870	33.46
3	Lu, Qian	University of Texas MD Anderson Cancer Center	20	1.40	276	13.80
4	Lumley Mark. A.	Wayne State University	20	1.40	469	23.45
5	Marx, Brian P.	VA Boston Healthcare System	20	1.40	365	18.25
6	Stanton, Annette L.	University of California, Los Angeles	18	1.26	897	49.83
7	Smyth, Joshua M.	Pennsylvania State University	13	0.91	1,529	117.62
8	Graham, Steve	Arizona State University	12	0.84	1,357	113.08
9	Boal, Adriel	University of North Texas-Denton	11	0.77	158	14.36
10	Maercker, Andreas	University of Zurich	10	0.70	224	22.40

Ranking by the number of publications. TP, Total publications; PTP, Percentage of the total publications; TC, total citations; AC, Average number of citations per publication.

VOSviewer statistics shows that among 4,084 authors in the sample data, 0.3% of them (10 authors) have published 10 or more documents. However, 114 authors, accounting for 2.8% of the total number, have received more than 100 citations. Table 4 lists the most productive and influential authors with at least 10 publications and over 100 citations.

According to the data, Pennebaker, with 40 articles, had the most publications, followed by Sloan (26 articles), Lu, Lumley, and Mark each with 20 articles. In citations, Pennebaker had a far larger number of citations than the other authors, even far more than the sum of the rest of the most productive authors in the field. He is the initiator of expressive writing studies in psychological science and the most influential researcher in the field. Besides, among the top 10 productive scholars, six of their respective affiliations were on the list of highly contributive institutions, namely University of Texas at Austin, University of California, Los Angeles, University of Texas MD Anderson Cancer Center, Wayne State University, VA Boston Healthcare System and Pennsylvania State University.

**Figure 4:**
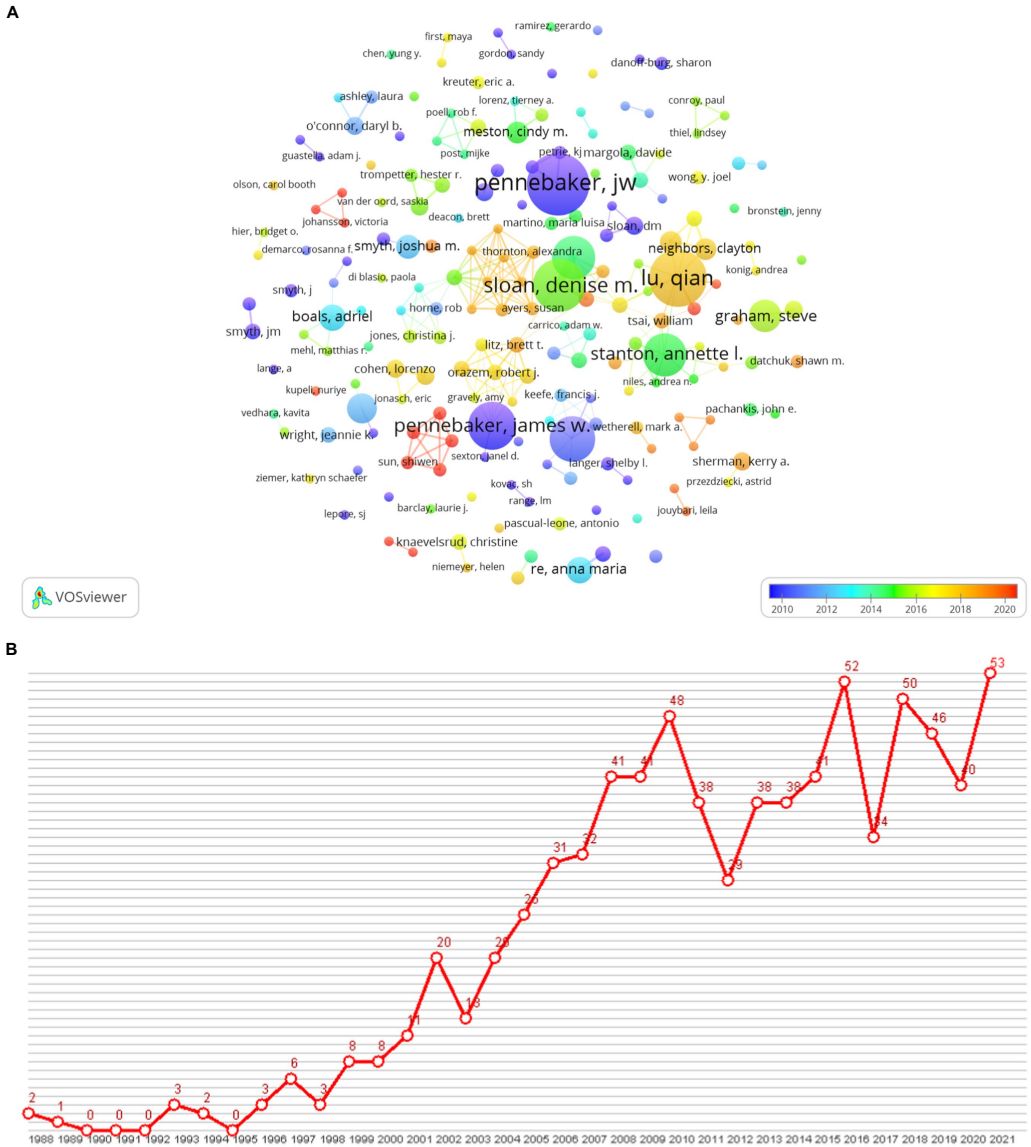
**(A)** Author cooperation network in expressive writing. In co-authorship function, the minimum number of documents of an author was set 3. A total of 197 authors met the thresholds and appeared in the map. When setting five items as the minimum cluster size, there were 83 clusters of author cooperation networks with the biggest network including 14 authors. **(B)** Author citation history graph of James Pennebaker.

The author cooperation network map (see Figure 4A) shows that the author collaboration in research on expressive writing was quite centralized. It can be observed that Pennebaker, Sloan, Lu, and Stanton had the most frequent academic collaborations. However, in the past 10 years, the collaboration of Pennebaker on the research of expressive writing occurred more frequently before 2014. Sloan and Stanton’s respective collaborative works took place in the middle of the last decade and Lu’s strong academic connections were more salient in recent years.

According to the author co-citation analysis in VOSviewer, James W. Pennebaker from the University of Texas at Austin is the top co-cited author among all, with a co-citation number of 2,822 and the total link strength being 57,504. Pennebaker initiated the expressive writing paradigm in conducting research in psychology in the 1980s. The author citation history of Pennebaker is demonstrated in Figure 4B. Although there were fluctuations, it can be observed that an increasing number of studies continue to cite Pennebaker’s work because of his ground-breaking contribution in using expressive writing in psychological investigations.

In conclusion, with regard to the publishing entities in expressive writing studies in terms of authors, institutions, and countries/regions, the most productive country is United States, with its highly contributive institution University of Texas at Austin where the most influential author James W. Pennebaker has been working.

#### Highly Contributive Journals and Disciplinary Distribution

**Figure 5:**
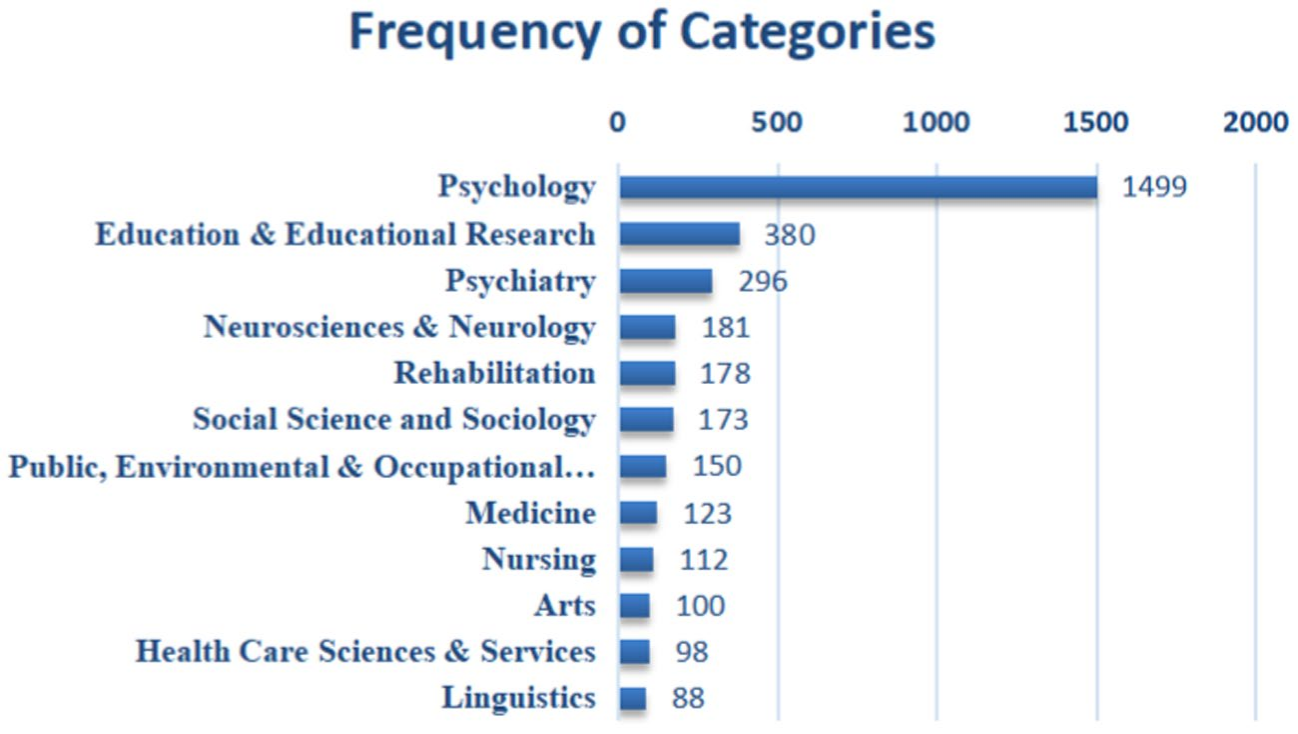
The top 12 categories/disciplines of expressive writing studies.

Among 1,429 literature in the field of expressive writing, a total of 279 categories were covered according to the data from CiteSpace. The distribution of the top 12 disciplines/categories is shown in Figure 5. The top 3 disciplines shown in Figure 5 were psychology, education, and psychiatry. To be more specific, in psychology, social psychology ranked the highest in the frequency of categories (449 times), followed by multidisciplinary practices in psychology (222 times), psychoanalysis (133 times), experimental psychology (71 times), educational psychology (49 times) and clinical psychology (33 times). The distribution of categories suggests that although expressive writing studies started in psychology and even now it is still investigated predominantly in psychological science, a diversity of disciplines have been involved in the research.

The 1,429 publications collected were published in 732 journals based on VOSviewer statistics, suggesting that this research topic has been to the interest of various publication sources of different fields although its main area is in psychology and health. Since the impact of each journal is assessed by the number of articles published by the journal in the field and the number of citations the journal holds (Dzikowski, 2018), Table 5 presents the most influential journals in the data collection with 10 or more publications and with more than 200 citations. The ranking is in accordance with the order of the number of citations. It can be observed from Table 5 that 4 out of the 13 most productive journals in expressive writing are journals focusing on “Health,” indicating that the studies of expressive writing are related to people’s physical and psychological health. However, the top ranking in publication number shows that these journals are the ones showing more interest in studies related to expressive writing. *British Journal of Health Psychology* and *Psychology & Health* each published 25 articles, surpassing other journals. It is also worth nothing here that *Journal of Poetry Therapy,* even though it is an ESCI-indexed journal, ranked third (22 articles) among all the journals publishing articles on expressive writing.

**Table 5:** The top 13 most published journals in expressive writing studies.

Rank	Journal	TP	PTP (%)	IF2020	TC	AC
1	British Journal of Health Psychology	25	1.75	3.311	571	22.84
2	Psychology & Health	25	1.75	3.073	583	23.32
3	Journal of Social and Clinical Psychology	19	1.33	1.946	458	24.11
4	Journal of Consulting and Clinical Psychology	17	1.19	5.348	3,048	179.29
5	Health Psychology	16	1.12	4.267	782	48.88
6	Psychosomatic Medicine	15	1.05	4.312	830	55.33
7	Journal of Health Psychology	13	0.91	3.231	237	18.23
8	Behaviour Research and Therapy	12	0.84	4.473	985	82.08
9	Cognitive Therapy and Research	12	0.84	2.503	244	20.33
10	Journal of Clinical Psychology	12	0.84	2.885	889	74.08
11	Journal of Traumatic Stress	12	0.84	3.476	246	20.50
12	Cognition & Emotion	11	0.77	2.678	704	64.00
13	Journal of Language and Social Psychology	10	0.70	2.253	2,217	221.70

TP, Total publications; PTP, Percentage of the total publications; IF, Impact Factor; TC, total citations; AC, Average number of citations per publication.

Since Impact Factor (IF) is a prevalent indicator of the influence and quality of academic journals (Garfield, 2006). Table 5 included IF 2020 provided by Journal Citation Reports (JCR) to exhibit the academic level of the journals publishing articles in the area. Table 5 shows that *Journal of Consulting and Clinical Psychology* (IF 5.348), *Behaviour Research and Therapy* (IF 4.473), *Psychosomatic Medicine* (IF 4.312) have the top 3 highest Impact Factor among all the journals. Since citation analysis is also an important indicator of the influence and performance of journals and publications. Average citations per article (AC) was adopted to demonstrate the breadth of the audience and the influence of the related publications. The result shows that *Journal of Language and Social Psychology* (AC = 221.70) has the most citations. It should be noted here that Tausczik and Pennebaker’s “The Psychological Meaning of Words: LIWC and Computerized Text Analysis Methods” is the most cited article in the journal and the article alone has 2,045 citations. Other journals actively cited by scholars were *Journal of Consulting and Clinical Psychology* (AC = 179.29), *Behaviour Research and Therapy* (AC = 82.08), and *Journal of Clinical Psychology* (AC = 74.08). Researchers who are doing research in expressive writing could use this list of journals for submissions or to search for influential studies.

### Content Analysis

#### Highly Cited Articles

Highly cited papers have been regarded as an important indicator in identifying excellent scientific research (Aksnes, 2003), and the number of citations can reflect the influence and popularity of certain literature. Thus, the analysis of the highly cited publications gives readers and researchers an overview of the focus of the research area. The basic information of the top 11 cited articles in the field of expressive writing is listed in Table 6.

**Table 6:** The top 11 cited papers in expressive writing studies.

Author	Title	Journal	Year	TC
Tausczik and Pennebaker JW	The psychological meaning of words: LIWC and computerized text analysis methods	Journal of Language and Social Psychology	2010	2045
Pennebaker JW	Writing about emotional experiences as a therapeutic process	Psychological Science	1997	1,148
Frattaroli J	Experimental disclosure and its moderators: a meta-analysis	Psychological Bulletin	2006	755
Smyth J	Written emotional expression: Effect sizes, outcome types, and moderating variables	Journal of Consulting and Clinical Psychology	1998	752
Pennebaker JW, et al.	Disclosure of traumas and immune function—health implications for psychotherapy	Journal of Consulting and Clinical Psychology	1988	741
Pennebaker JW and Seagal	Forming a story: the health benefits of narrative	Journal of Clinical Psychology	1999	692
Graham S	A meta-analysis of writing instruction for adolescent students	Journal of Educational Psychology	2007	659
Pennebaker JW and Francis	Cognitive, emotional, and language processes in disclosure	Cognition & Emotion	1996	566
Pennebaker JW, et al.	Linguistic predictors of adaptive bereavement	Journal of Personality and Social Psychology	1997	546
Pennebaker JW	Putting stress into words—health, linguistic, and therapeutic implications	Behaviour Research and Therapy	1993	539
Smyth J, et al.	Effects of writing about stressful experiences on symptom reduction in patients with asthma or rheumatoid arthritis—a randomized trial	Jama-Journal of The American Medical Association	1999	470

List represented in CiteSpace. TC, total citations.

Almost all top-cited papers were published in earlier years and all were from the scholars in the United States. An overwhelming majority of them, 8 out 11 (72.7%), were published in 1990s, and only three were published between 2000 and 2010. The first three top-cited articles were from *Journal of Language and Social Psychology*, *Psychological Science,* and *Psychological Bulletin*, two of them were the leading journals in psychology. This result echoed the finding that highly cited papers were published primarily in high-impact journals (Aksnes, 2003). Also, similar to findings of Aksnes (2003) in identifying the characteristics of highly cited papers, review articles are more likely to be present in highly cited papers, and in this case around 27% of the top-cited papers are reviews, such as the studies of Frattaroli (2006); Smyth (1998), and Graham and Perin (2007). It is also easy to observe that among the top 11 most cited articles, seven (63.6%) were from Pennebaker, the most influential leading scholar in the studies of expressive writing.

The citations of the first top-cited article of Tausczik and Pennebaker (2010) (TC = 2,045) far exceeded citations of other papers, because the two researchers created a computerized text analysis method to do linguistic analysis of written texts by calculating psychological indicators to reveal the participants’ personality, emotions, etc. Thus, a large number of researchers around the world were attracted to this new tool to analyze texts. Up to now, 312 articles were published with the keyword “LIWC” (Linguistic Inquiry and Word Count), the name of the software.

The second most cited article is by Pennebaker (1997) (TC = 1,148), “Writing about emotional experiences as a therapeutic process” published in *Psychological Science*. It summarized the basic writing paradigm that is widely used by other researchers. Some variables affecting disclosure effects were discussed in the article, such as the effects of talking about traumatic events vs. writing about them, the choice of topics in written disclosure, the frequency of the writing sessions, feedback to participants, individual difference, such as sex, age, or anxiety level, and linguistic or cultural factors. Pennebaker also reported the analyzed results of linguistic factors in the written disclosure and found that people who benefit more may be the ones whose writing “began with poorly organized descriptions and progressed to coherent stories”(Pennebaker, 1997, p. 165). Thus, one’s verbal way of presenting their ideas can reflect their psychological state, and obvious changes in language and textual presentation could happen if expressive writing as an intervention was employed.

The third top-cited paper is by Frattaroli (2006) (TC = 755), published in *Psychological Bulletin*. In her study “Experimental Disclosure and Its Moderators: A Meta-Analysis,” Frattaroli examined 146 studies of experimental disclosure, with different variables as moderators, such as sample size, disclosure instructions, the number of writing sessions, the frequency spacing of writing sessions, and the timing of the follow-up evaluations, along with the effect size. The study found that exposure theory proves most effective among different theoretical attempts in explaining the positive health outcomes of expressive writing. Mixed support is found for other theories, such as disinhibition theory, cognitive processing theory, self-regulation theory, and social integration theory.

The fourth most cited reference is by Smyth (1998) “Written Emotional Expression: Effect Sizes, Outcome Types, and Moderating Variables” (TC = 752), published in the *Journal of Consulting and Clinical Psychology*. The study reported four dimensions of possible enhanced health outcomes resulting from expressive writing intervention: physical health, psychological wellbeing, physiological functioning, and general functioning. Unlike Smyth (1998) whose subjects were healthy participants, in the study of Frisina et al. (2004), the participants were people with physical and psychiatric symptoms. After meta analyzing nine studies, it is found that expressive writing creates more beneficial effects on physical than psychological health, and the health effect of expressive writing on people with clinical disorders is less robust than the benefits it has on healthy people.

#### High-Frequency Keywords

Keywords are highly condensed words to summarize the core content of a document. Keyword frequency can be used to detect changes in themes (Chen, 2004). In previous studies, some researchers used occurrence frequency or co-occurrence frequency of keywords to investigate the major research domains in literature and generated clusters from the pattern of co-occurrences to study the interaction between scientific literature and the evolution of research in different subjects (Callon et al., 1991; Law and Whittaker, 1992). In order to better understand the research domains from a horizontal perspective in expressive writing, this study employed frequency of co-occurrence of author keywords to reveal the research focus in the publication dataset. In VOSviewer, we chose “co-occurrence” as type of analysis and “author keywords” as unit of analysis and used “full counting” as the counting method, and the result showed that In the 1,429 articles, there were 2,812 author keywords. When the minimum number of occurrences of a keyword was set 10 times, there were 58 keywords left, with the “expressive writing” appeared 311 times (occupying 11.01% of all the keywords). Then after 12 searching items/words/phrases were manually excluded from the list including “expressive writing,” “writing,” “trauma,” “disclosure,” “intervention,” “emotional disclosure,” “writing intervention,” “emotion,” “emotional expression,” “writing therapy,” “written disclosure,” and “written emotional disclosure,” the top 30 high-frequency keywords were shown in Table 7.

**Table 7:** The top 30 high-frequency author keywords in expressive writing studies.

Rank	Frequency	Keywords	Rank	Frequency	Keywords
1	62	Depression	16	19	Coping
2	55	Stress	17	19	Creative writing
3	33	Anxiety	18	19	Wellbeing
4	32	PTSD	19	17	Self-compassion
5	30	Narrative	20	17	Therapeutic writing
6	27	Mental health	21	16	Emotional processing
7	27	Quality of life	22	16	Qualitative
8	26	Cancer	23	15	Randomized controlled trial
9	26	Posttraumatic stress disorder	24	15	Resilience
10	26	Psychotherapy	25	14	Bereavement
11	25	Breast cancer	26	14	Chronic pain
12	22	Health	27	14	HIV
13	21	Meta-analysis	28	14	Self-disclosure
14	20	emotion regulation	29	14	Social support
15	19	Adolescents	30	13	Emotions

It can be seen from the list that the most researched topics are related to psychological issues, such as depression, stress, and anxiety, the top 3 high-frequency keywords the authors of the articles adopted. Health is another key issue in academic discussion, as displayed by the keywords, such as mental health, cancer, breast cancer, chronic pain, and HIV. It is interesting to note that creative writing and therapeutic writing were also discussed together with expressive writing in psychological explorations. This might suggest a future research potential that these two types of writing could be used jointly or comparatively for therapy studies.

#### Keyword Co-occurrence Analysis

This section adopted 4,766 “All keywords,” including author keywords and keywords plus provided by WoS as units of analysis. Suggested by study of Chen et al. (2016), keywords with frequencies lower than a certain number implied that fewer researchers paid attention to them. Thus the threshold of the minimum number of occurrences of a keyword was set 10, and 218 words met the threshold. Further, there was a manual exclusion of 16 searching items and keywords, such as “disclosure,” “expressive writing,” and “emotional disclosure”, etc., ultimately leaving 202 keywords for analysis and co-occurrence map generation.

**Figure 6:**
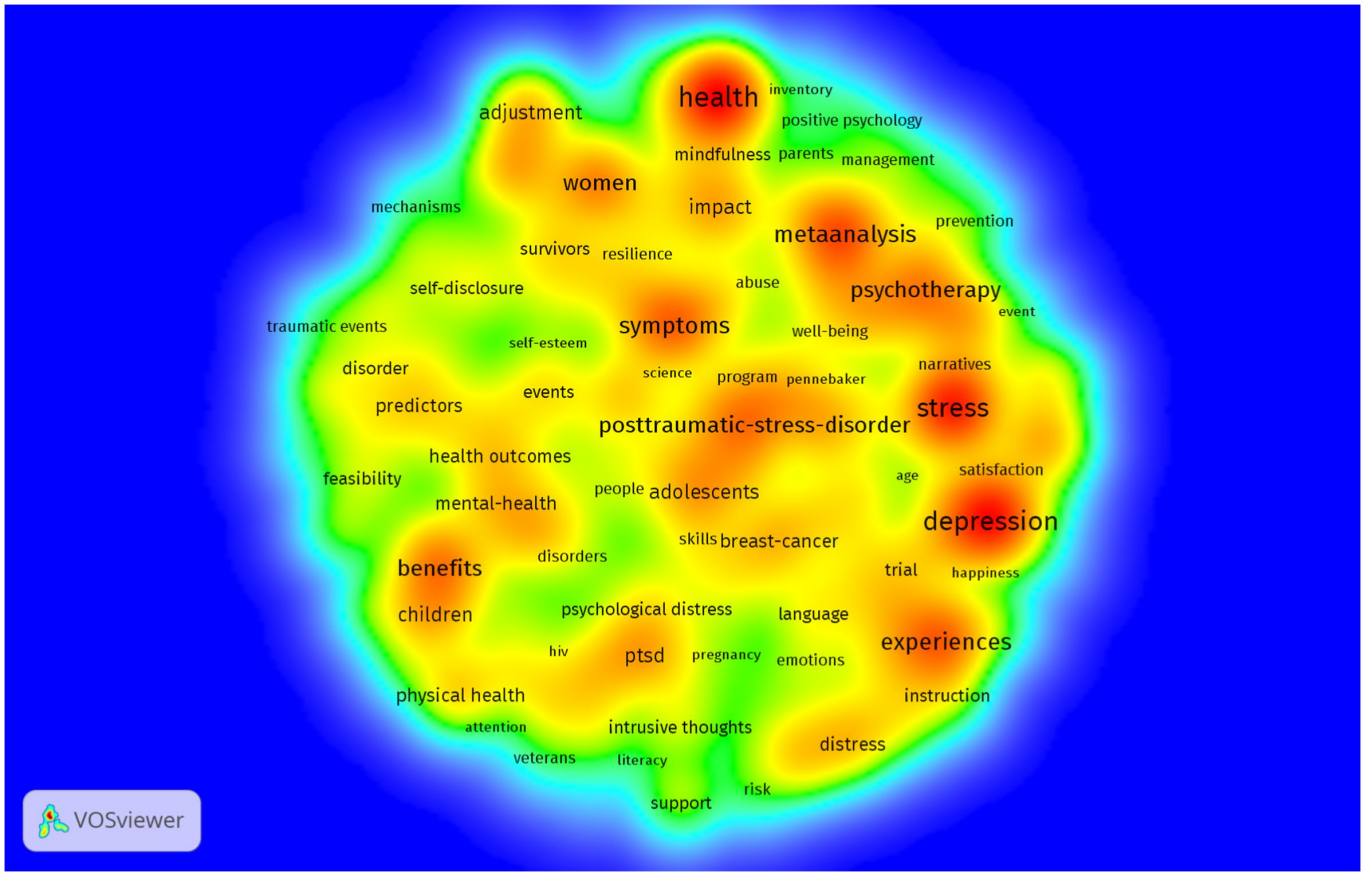
Heat map of density visualization of keywords co-occurrence network. Visualization scale weights were based on total link strength. Layout setting: attraction = 5, repulsion = −6. The area in red represents higher frequency of related keywords, the yellow or red-yellow areas suggesting less heated keywords discussed by scholars and the green areas representing even less density and lower degree of heat in research.

As illustrated in Figure 6, density measures the inner strength of different keywords and connections between them (Rojas-Lamorena et al., 2022). The shades of the colors represent the high or low density of the keyword network. The map shows that the most focused research directions in expressive writing were the dark red areas, covering health, stress, and depression, which were in line with the findings in high-frequency keywords analysis. Other keywords having also attracted researchers’ interest were symptoms, posttraumatic stress disorder (PTSD) especially for adolescents, psychotherapy, and the benefits of expressive writing. It could also be observed from the red-color keyword cluster in the upper-right corner of Figure 6 that meta-analysis might be one of the popular ways to do research in this area.

## Discussion

### Thematic Analysis: Subtopics Categorization

**Figure 7:**
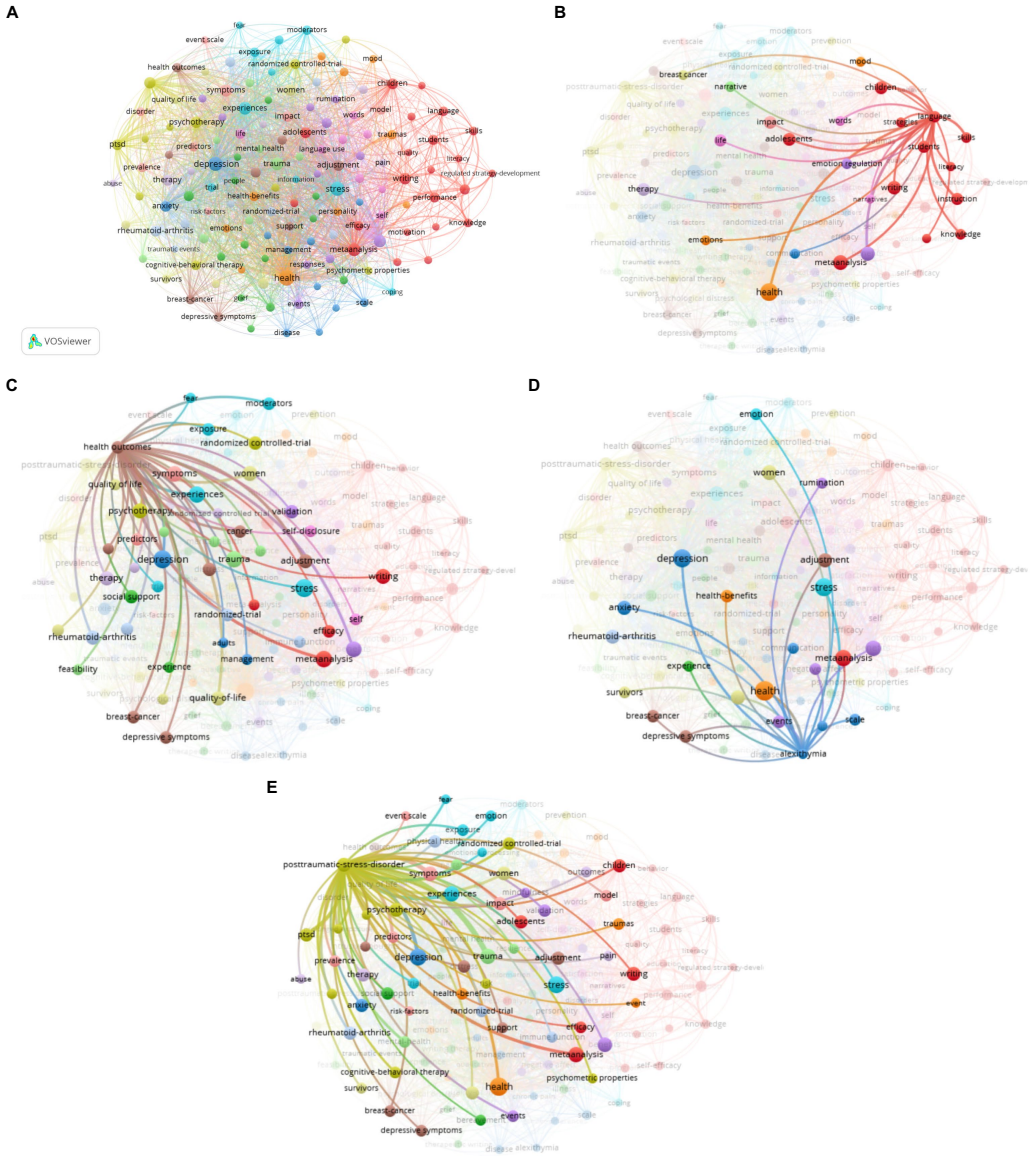
**(A)** Results of co-word analysis. Excluding searching keywords/items: disclosure, expressive writing, emotional disclosure, intervention, written emotional expression, expression, written emotional disclosure, emotional expression, written disclosure, writing, interventions, expressive writing intervention, and writing intervention. Layout setting: attraction = 2, repulsion = −3. **(B)** Relationship map between language and other keywords. **(C)** Relationship map between health outcomes and other keywords. **(D)** Relationship map between alexithymia and other keywords. **(E)** Relationship map between posttraumatic stress disorder (PTSD) and other keywords.

Co-word analysis, similar to topic modeling, is a powerful technique to identify and represent the thematic focus from a population of papers. In this study, co-word analysis is to reveal the association strength between the topical terms that have drawn researchers’ attention represented in the relevant publications in the field of expressive writing studies. There are several steps involved for co-word analysis: extracting keywords from author keywords and keywords plus, selecting high-frequency terms and excluding searching terms, generating co-occurrence map, clustering topical keywords to theme-based knowledge map, and interpreting the research themes. Among 4,766 keywords, 153 keywords met the threshold when the minimum number of occurrences of a keyword was set 15. Figure 7A illustrated keyword map clusters based on the connections between 153 extracted keywords.

Cluster 1 in red includes 22 items. This cluster includes keywords such as regulated strategy development, adolescents, behavior, efficacy, instruction, language, knowledge, literacy, and working memory. The connection between the focal term “language” and other keywords is presented in Figure 7B.

Numerous studies have been conducted with regards to youngsters’ language performance in their expressive writings. Re et al. (2007) conducted a research on children who were reported to be exhibiting symptoms of attention-deficit/hyperactivity disorder (ADHD) and found that these children have poor expressive writing skills. The difficulties in writing can also be found in other different types of tasks and age groups in school. Re et al. (2008) continued their previous study to improve the concentration ability of these children with ADHD by using some guided practices preceded by a brief training. As well, Carretti et al. (2013) conducted a comparative research between good and poor comprehenders and found that the issues of using coherence and causality in narratives texts will make poor comprehenders achieve a lower score although the two groups have similar performance in descriptive texts. Most research within this cluster was conducted to examine the participants’ linguistic features to predict research outcomes by the use of expressive writing because the “benefits of writing are predicted by emotional and cognitive characteristics of the writings” (Warner et al., 2006, p. 558). Furthermore, Warner et al. (2006) in their study used language as a predictor of health outcomes, and the result revealed that expressive writing improves the emotional and behavioral functioning in adolescents with asthma.

Cluster 2 has 13 keywords, led by “health outcomes,” including bereavement, care, creative writing, experience, grief, HIV, identity, illness, people, qualitative, social support, therapeutic writing, and writing therapy, each keyword in close connection with different health issues (See Figure 7C).

A lot of studies have proved the positive health outcomes obtained from the practice of expressive writing. Study participants who have written their thoughts and feelings about traumatic or stressful experiences have often shown improvements in their physical health (Pennebaker, 1997; Lepore et al., 2002) and fewer health clinic visits (Pennebaker, 1993; Esterling et al., 1999; King, 2016). Meanwhile, disclosure of one’s emotional experiences can be represented in different forms, such as visual arts and “movement-based creative expression” (Stuckey and Nobel, 2010), apart from expressive writing itself which can also take a variety of text forms including journaling and poetry writing in the art of healing (Coulehan and Clary, 2005). However, even though written emotional disclosure has been reported to have physical and psychological benefits (Frattaroli, 2006), the studies show that it has mixed results. For example, in the study by Rosenberg et al. (2002), it is found that written emotional disclosure does not provide a positive impact for physical symptoms in the disease-relevant aspects of a cancer population.

According to Pennebaker, there is no fixed single theory that can adequately explain why the expressive writing paradigm benefits people’s health and mood, as the positive effects are observed on multiple levels, such as “cognitive, emotional, social, and biological” improvements (Pennebaker, 2004, p. 138). For instance, studies validated that expressive writing can promote health in varied ways and in different group of people, such as HIV patients (Petrie et al., 2004) and Fibromyalgia patients (Gillis et al., 2006). More related studies need to be conducted to see how expressive writing works among different populations in different situations.

Cluster 3 holds 11 items and could be labeled as “psychological issues,” including keywords such as adults, alexithymia, anxiety, chronic pain, communication, depression, disease, disorders, gender differences, management, etc.

The example relationship map (See Figure 7D) reveals that researchers who are interested in alexithymia might also pay attention to depression, anxiety, stress, and related emotional problems. There are studies that use expressive writing both in experimental groups and control groups. For example, in “Benefits of Expressive Writing in Lowering Rumination and Depressive Symptoms” by Gortner et al. (2006), depression-vulnerable university students participating in an expressive writing study showed much lower depression symptoms 6 months after the experiment, compared to that of the control group. Meanwhile, Graf et al. (2008) examined the effectiveness of expressive writing as homework for outpatient psychotherapy clients by using Pennebaker’s writing paradigm. The results suggest that the experimental writing group showed significant decrease in symptoms of anxiety and other mental health symptoms. At the same time, other studies have shown that compared to traditional expressive writing control groups, the experimental self-compassion writing group reduced shame-proneness and negative affects (Johnson and O’Brien, 2013) and had a greater improvement on mood (Odou and Brinker, 2014). It is also interesting to note that in this cluster, gender and gender difference (see the keyword “women” in the map) emerged as an important element bringing observable differences in the intervention results. In “Effects of expressive writing on depressive symptoms,” Reinhold et al. (2018, p. 9) found that the “female participants appeared to benefit more.”

Cluster 4 and 5, each having 11 items, are about using effective measures to address emotional issues. Cluster 4 includes keywords, such as PTSD, cognitive behavioral therapy, prevention, prolonged exposure, psychometric properties, psychotherapy, risk, and veterans. Cluster 5 includes keywords such as benefits, emotion regulation, mindfulness, and self-compassion. Figure 7E displays the relationship map between PTSD and other keywords.

Sloan and Marx (2004) found that expressive writing was an effective measure to reduce depressive symptoms in women with PTSD symptoms. Other measures to reduce the emotional issues are expressive group therapy, social support, and self-help, to help people make psychological adjustments to overcome posttraumatic stress and promote posttraumatic growth.

Recent studies also show that Asian Americans and Chinese-speaking population, once overlooked in the field, have also become participants in research. This addition is attributed to the studies conducted by Qian Lu whose research interests include cultural diversity and Asian Americans. Lu’s goal in research is to promote health and wellbeing through a cultural lens, as well as through a biopsychosocial lens. Her research interests are in cultural health psychology, cultural sensitivity for social support intervention, and Asian Americans. In Lu et al. (2012) “A pilot study of expressive writing intervention among Chinese-speaking breast cancer survivors,” the participants of this research were specifically Chinese-speaking people, since few prior studies “focused on Asian American breast cancer survivor’s psychological needs.” (Lu et al., 2012, p. 548). The research found that expressive writing has potential benefits for Chinese-speaking breast cancer survivors in improving their long-term health outcomes, such as “quality of life, fatigue, posttraumatic stress, intrusive thoughts, and positive affect” (Lu et al., 2012, p. 548). It is suggested that written emotional disclosure can be employed as one of the strategies in supporting cancer survivors who speak languages other than English, especially in the American cultural context.

This change in research focus can also be a sign for future research opportunities in expressive writing. For instance, being inclusive of participants speaking different languages provides a space for future studies to explore the possibilities of second-language or foreign language expressive writing. Moreover, it would be a valuable addition to the current scientific literature to investigate the effects of bilingual or multilingual expressive writing on the health outcomes of immigrants who are facing cultural challenges when living in a new environment in another country. Besides, thematic analysis indicates that future research trends in expressive writing might be motivated by the thriving of positive psychology and more studies on positive expressive writing are expected.

### Thematic Evolution: Burst Terms Detection

**Figure 8:**
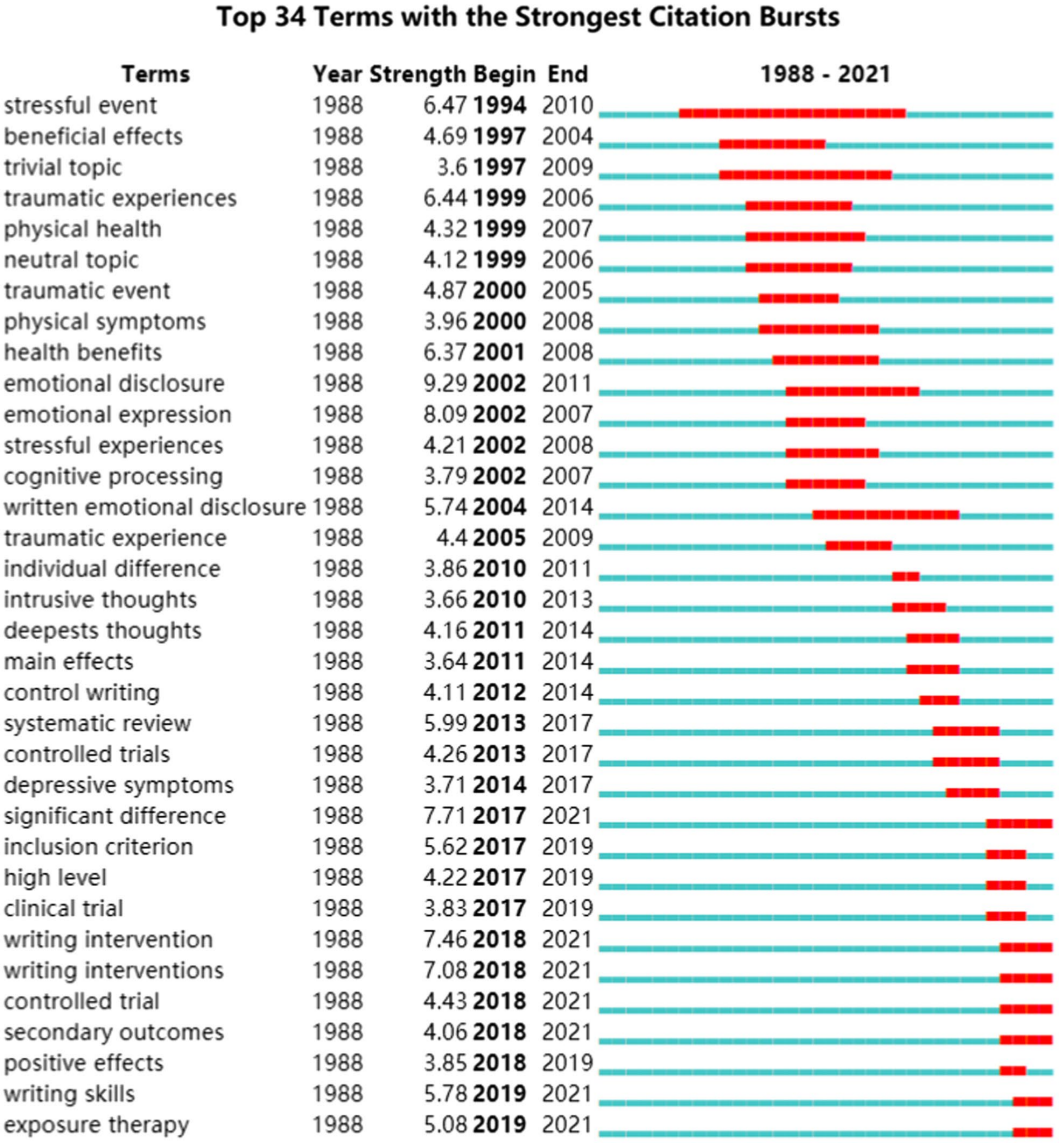
Thematic evolution based on the top 34 terms with strongest citation bursts. Note: “Begin” and “End” suggest the period when activities such as publications or citations were most intense. Manually excluding the searching terms: expressive writing, writing intervention, emotional disclosure, expressive writing intervention, emotional expression, writing intervention, written emotional disclosure, writing therapy, expressive writing interventions, written disclosure, and written emotional expression.

Burst detection in CiteSpace can be used to reveal the sharp emerging increases of scientific interest in a specific area (Chen, 2006) and it can be used to explore the thematic trends of a field and current research front. The burstness can detect a sudden change in word frequency based on the Kleinberg algorithm (Kleinberg, 2003). Therefore, in order to analyze the longitudinal evolution process of studies on expressive writing, we conducted a thematic evolution analysis by using the term bursts function in CiteSpace. The scope from which terms were extracted were from title, abstract, author keywords, and keywords plus in a total 1,390 qualified records provided in CiteSpace using 1 year per slice. Figure 8 shows the ongoing bursts represented by the red lines.

As Figure 8 shows, the thematic evolution can be summarized according to three different periods.

Between 1994 and 2008 (see the column of the beginning year under the head “Begin”), the major terms were “stressful event,” “beneficial effects,” “traumatic experiences,” “physical health,” “health benefits,” and “cognitive processing.” It could be observed that the early studies mainly concerned about the benefits of expressive writing on health. Sufferers of different illnesses, such as rheumatoid arthritis and breast cancer, have gained positive benefits from written disclosure. For example, Creswell et al. (2007) found that self-affirmation writing as a form of expressive writing helped ease the psychological difficulties of early-stage breast cancer survivors.

Between 2010 and 2018, the interests of researchers were more diversified. There were more studies on “individual difference,” “intrusive thoughts,” and “depressive symptoms.” Actually, the studies of intrusive thought started over two decades ago. Klein and Boals (2001) found that expressive writing decreased intrusive thoughts and avoidant thinking about stressors, which results in gaining larger working memory. Lu et al. (2015) discussed the influence of individual differences in optimizing the effect of expressive writing intervention. Rude and Haner (2018) argued for in important role of individual differences in discussing the impact of expressive writing on depressive symptoms.

In more recent years after 2018, “secondary outcomes,” “positive effects,” and “exposure therapy” have become the hot spots of research. It is also interesting to note that (random) controlled trial (RCT) was another hot term used by recent studies, since researchers now have started to adopt RCT as the main empirical research method instead of using the term “experiments”. Besides, Due to the rapid development of positive psychology, the research focusing on stress, trauma, and pain seemed to have been redirected to the “positive effects”, such as “posttraumatic growth,” “quality of life,” and “resilience.” Because of the pandemic, “COVID-19” has been included in studies in the past two years. Bechard et al. (2021) found that the online expressive writing intervention in the general population proves effective in improving resilience during the COVID-19 pandemic. Davis et al. (2020) proposed that expressive writing can promote athletes’ mental health due to disruptions to training sessions caused by the COVID-19 pandemic. Thus, the application of expressive writing in new circumstances, such as helping people reduce the negative impact created by the pandemic demonstrates its practical value and future potential of using expressive writing in different fields and situations.

## Limitations

Like all scientific research, this study has some limitations. Firstly, the sample bibliographic records were retrieved only from Web of Science Core Collection. Although this database is the most recognized, and includes most of the influential databases, and has journals with greatest scientific impact from different subjects and categories, the present study does not capture all the publications on the topic because there are other academic publications on expressive writing beyond WoS. Therefore, it should be cautious when generalizing the results. Future related studies would extract records from other scientific databases, such as Scopus and Google Scholar, in order to collect publications not indexed in WoS. Secondly, in order to decrease the rate of the replicated content in the references and to ensure the high-quality of the papers, we restricted the data collection to two document types, articles, and reviews. Further research can extend the document types to other scientific publications, such as conference proceeding papers, books, and book chapters, which may provide more insights and slightly different findings. However, these type of articles would present challenge in data analysis since they might not be high-quality peer-reviewed publications (Rojas-Lamorena et al., 2022) or they do not include essential bibliographic records in bibliometric approach. Thirdly, the searching strategy might not be able to include all the relevant scientific publications although different searching keywords were applied. Although the starting year of searching was set in 1981, the seminal paper of Pennebaker and Beall (1986) was not obtained even though the article was the first one in the field because the terms, such as “expressive writing,” “written emotional disclosure,” and other terms, were only adopted years later after the first experiment was conducted by Pennebaker and Beall. Future research can use varying searching strategy so that the database of analysis could be more inclusive. A further limitation is related to the applied tools themselves. Although bibliometric analysis using software is efficient in providing a quantitative analysis and holistic visualized landscape of an academic field, it does not guarantee that the tools were completely accurate in their mathematical calculation. When functioning different types of analysis, the tools may eliminate certain papers with marginal importance to the topic due to different calculation method inside the software. Besides, adopting varied thresholds and parameters can yield different mapping structures even though there are no significant changes in the results.

Regarding potential future research deriving from this study, other bibliometric techniques can be used, such as co-citation analysis of the references and co-word analysis of funded projects. By using bibliometric method, it can be expected that there are enormous opportunities for further applications to explore other focal topics in this area.

## Conclusion

This study is the first systematic quantitative and visualized analysis of the research on expressive writing. It employed a bibliometric method and scientific knowledge mapping, with the combined use of VOSviewer and CiteSpace, to present all the articles published in almost 40 years and indexed in the core collection of Web of Science database. This study detailed the trend of the publications and analyzed the publication performance of the productive authors, institutions, and countries/regions. In order to help the reader better understand the dynamic development of the area, the study further conducted reference keyword analysis, thematic analysis, and topic evolution in the hopes of bringing deeper insights to the overview of this academic area. This panoramic view should assist scholars in understanding the development of expressive writing research in a holistic way, and enable potentially interested researchers to grasp the current status of the field with high efficiency before they embark on their own studies.

## Data Availability Statement

The raw data supporting the conclusions of this article will be made available by the authors, without undue reservation.

## Author Contributions

The author confirms being the sole contributor of this work and has approved it for publication.

## Funding

This work was supported by the 11th China Foreign Language Education Fund (Grant Number ZGWYJYJJ11A137); Fundamental Research Funds for the Central Universities (Grant Number 2021YQWF03); the Higher Education Research Project in Coal Industry (Grant Number 2021MXJG174); and National Social Science Foundation of China (Grant Number 20BZW174).

## Conflict of Interest

The author declares that the research was conducted in the absence of any commercial or financial relationships that could be construed as a potential conflict of interest.

## Publisher’s Note

All claims expressed in this article are solely those of the authors and do not necessarily represent those of their affiliated organizations, or those of the publisher, the editors and the reviewers. Any product that may be evaluated in this article, or claim that may be made by its manufacturer, is not guaranteed or endorsed by the publisher.
